# Antineoplastic properties and pharmacological applications of *Crotalus durissus terrificus* snake venom

**DOI:** 10.1590/0037-8682-0323-2022

**Published:** 2022-12-16

**Authors:** Brunna Fernanda Arraez Alves, Rui Seabra Ferreira

**Affiliations:** 1 Faculty of Animal Science and Food Engineering, University of São Paulo, Pirassununga, SP, Brazil.; 2 Center for the Study of Venoms and Venomous Animals (CEVAP), São Paulo State University (UNESP), Botucatu, SP, Brazil.

**Keywords:** Snake Venom, Cancer, Antitumor, Crotalid Venoms, Crotalus

## Abstract

Snake toxins are widely studied owing to their importance in snakebite accidents, a serious public health issue in tropical countries, and their broad therapeutic potential. Isolated fractions from venom produced by snakes of the genus *Crotalus sp.* present a wide variety of pharmacological uses such as antifungal, antiviral, antibacterial, and antitumor properties, among other therapeutic potentialities. Given the direct effect of this venom on tumor cells, isolation of its compounds is important for the characterization of its anticarcinogenic actions. *Crotalus durissus terrificus* venom and its toxins have been widely evaluated as potential candidates for the development of new antineoplastic therapies that are efficient against different tumor lines and cellular targets. This review highlights the venom toxins of this species, with a focus on their antineoplastic properties.

## INTRODUCTION

Currently, approximately 11,341 reptile species are recognized worldwide^1^, with 1,116 species found in Australia, 974 in Mexico, and 830 in Brazil. *Crotalus* comprises of the venomous *Viperidae* snakes^2^ ^-^ ^7^ from the subfamily *Crotalinae*, also known as rattlesnakes. These are distributed across South America, mainly from Colombia to Argentina^5^ ^,^ ^7^ ^-^ ^9^, with the following six subspecies found in Brazil: *Crotalus durissus cascavella, C. d. collilineatus, C. d. terrificus, C. d. marajoensis, C. d. ruruima,* and *C. d. durissus* ^4^ ^,^ ^6^ ^,^ ^9^. 

**FIGURE 1:**
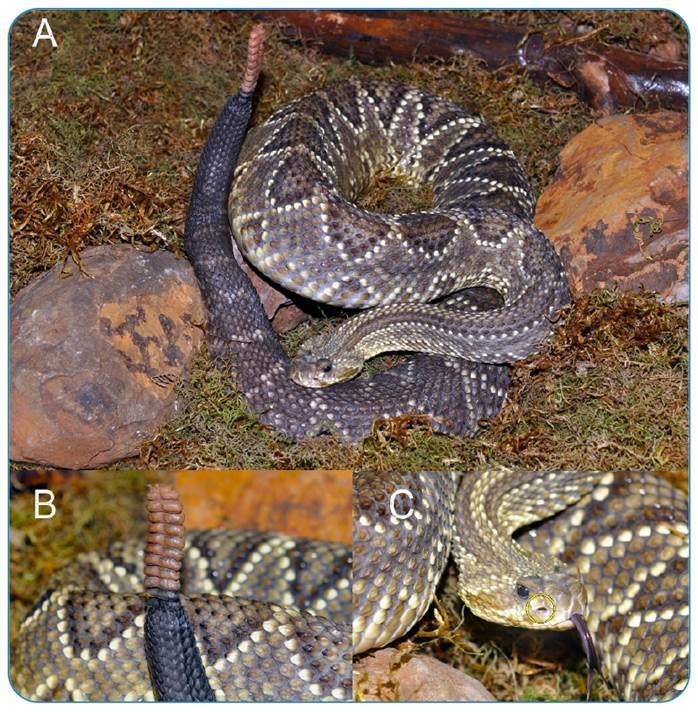
**(A)** *Crotalus durissus terrificus*; **(B)** Rattle detail; **(C)** Loreal Pit (yellow circle).

These snakes are primarily nocturnal^5^ and solenoglyphic dentition^5^ ^,^ ^10^ presents loreal pits, a thermoreceptor organ of viperid species, visible as openings between the eye and the nostril of the animal head, which are of great importance for the detection of temperature variations, particularly of prey and predators^5^. The most striking characteristic of *Crotalus* snakes is the presence of a rattle at the end of their tails (Figure 1)^5^ ^,^ ^6^. 

*Crotalus* snakes cause frequent and severe accidents, represent a serious public health problem in tropical countries, and the snakebites are considered a neglected disease by the World Health Organization^6^ ^,^ ^11^ ^-^ ^13^. However, venom is an important biotechnological tool because of the specialization and efficiency of its components, which affect a large number of targets with high selectivity and affinity^14^ ^-^ ^16^. 

## *CROTALUS DURISSUS TERRIFICUS* VENOM: COMPOSITION, GENERAL PHARMACOLOGICAL ACTIONS AND ANTINEOPLASTIC APPLICATIONS

Snake venom is one of the richest sources of bioactive substances in nature and is therefore of great interest for the development of new drugs^4^ ^,^ ^14^ ^-^ ^28^. Snake venoms are composed of a mixture of proteins, organic compounds, inorganic ions, carbohydrates, lipid fractions, and other substances^4^ ^,^ ^14^ ^,^ ^16^ ^,^ ^17^ ^,^ ^20^ ^,^ ^21^ ^,^ ^27^ ^,^ ^29^ ^,^ ^30^.

Proteins account for approximately 90% of the dry weight of snake venom^4^ ^,^ ^21^ ^,^ ^29^ ^,^ ^31^ ^,^ ^32^. *C. d. terrificus* (Cdt) venom is mainly composed of phospholipase A_2_ (PLA_2_), serinoproteases, hyaluronidases, L-amino acid oxidases, peptides, low molecular weight organic compounds, inorganic ions, and enzyme inhibitors^4^ ^,^ ^33^. The main toxins found in Cdt venom are Crotoxin, Convulxin, Gyroxin, and Crotamine^4^ ^,^ ^6^ ^-^ ^8^ ^,^ ^34^ ^-^ ^38^. This venom also contains more than 60 different protein families^23^. Envenomation generates local manifestations of pain, edema, erythema, paresthesia, and systemic manifestations such as eyelid ptosis, facial muscle paralysis, and myoglobinuria, among other clinical signs^4^ ^,^ ^6^ ^,^ ^25^ ^,^ ^31^ ^,^ ^35^ ^,^ ^39^, because of its neurotoxic, coagulant, and myotoxic actions^4^ ^,^ ^25^ ^,^ ^31^ ^,^ ^33^ ^,^ ^35^.

There is a wide variety of pharmacological uses of the different fractions of *Crotalus sp*. venom, including antifungal, antiviral, antibacterial, antitumor, and antiprotozoal activities^4^ ^,^ ^15^ ^,^ ^26^ ^-^ ^28^ ^,^ ^37^ ^,^ ^40^ ^-^ ^43^. 

## CROTOXIN

Crotoxin represents approximately 40%-60% of the dry weight of the Cdt venom^4^ ^,^ ^8^ ^,^ ^19^ ^,^ ^33^ ^,^ ^36^ ^,^ ^42^ ^-^ ^44^ and is a potent neurotoxin formed by PLA_2_ and crotapotin, forming a complex of high toxicity^4^ ^,^ ^8^ ^,^ ^32^ ^,^ ^38^ ^,^ ^42^ ^-^ ^54^, and exhibits myotoxic, nephrotoxic, and cardiotoxic effects^4^ ^,^ ^37^ ^,^ ^38^ ^,^ ^43^ ^,^ ^44^ ^,^ ^46^ ^,^ ^48^ ^,^ ^50^. 

This neurotoxic action is mainly attributed to the inhibitory mechanism of acetylcholine release in presynaptic neurons^48^ ^,^ ^52^ ^,^ ^54^. Desensitization of postsynaptic nicotinic receptors is another mechanism that reduces the response to acetylcholine^48^ ^,^ ^52^. Thus, crotoxin acts by blocking potassium channels and prolonging the action potential at neuromuscular junctions, thereby increasing calcium influx into the channels, mainly due to the presence and high activity of PLA_2_ in its composition^8^ ^,^ ^48^ ^,^ ^52^.

Crotoxin has been widely studied for its immunomodulatory, anti-inflammatory, antitumor, antimicrobial, and analgesic actions^4^ ^,^ ^40^ ^,^ ^43^ ^,^ ^44^ ^,^ ^46^ ^,^ ^48^ ^,^ ^50^ ^-^ ^54^. *In vivo* studies have demonstrated its ability to inhibit the production of pro-inflammatory and anti-inflammatory cytokines from the injection of the toxin in rats, including IL-10, IL-4, IL-6, and tumor necrosis factor^36^ ^,^ ^43^. This immunomodulatory activity may be associated with the production of anti-inflammatory mediators via the lipoxygenase pathway, such as lipoxin A4 (LXA4), and the activation of formyl peptide receptors, in addition to its regulatory role in macrophages^36^ ^,^ ^43^ ^,^ ^44^ ^,^ ^51^.

*In vitro* and *in vivo* studies have described activating mechanisms of cell apoptosis in different cancer cell lines^19^ ^,^ ^47^ ^-^ ^51^ ^,^ ^53^ ^,^ ^55^ induced by cellular autophagy mechanisms^47^ ^,^ ^53^. Both cell death pathways activated by crotoxin (apoptosis and autophagy) can occur simultaneously or sequentially through mechanisms such as changes in mitochondrial membrane potential and release of intracellular cytochrome C. Another important factor related to the mechanism of action of crotoxin is its apparent selectivity for cells with high expression of epidermal growth factor receptors (EGFR) ^19^ ^,^ ^21^ ^,^ ^47^ ^,^ ^50^ ^,^ ^56^. 

The cytotoxic action on glioblastoma and benign pituitary adenoma cells was partially attributed to crotoxin, which is also cytotoxic to human mammary duct carcinoma and human lung adenocarcinoma cell lines^4^ ^,^ ^19^ ^,^ ^47^ ^-^ ^51^ ^,^ ^55^ ^,^ ^57^. The application of portions of the toxin to murine erythroleukemia cells demonstrated the potential to reduce the viability of the strain^38^. To observe cytotoxicity, the B subunit of crotoxin was separated from PLA_2_ and used alone^38^. 

The isolated crotoxin is cytotoxic to different cell lines, with different cell response^53^. The mechanisms evaluated involved changes in the mitochondrial membrane potential, release of cytochrome C, and activation of caspase-3, a protease essential for the process of cell apoptosis^47^ ^-^ ^50^ ^,^ ^52^ ^,^ ^53^ ^,^ ^55^. Furthermore, it was possible to conclude that the toxin did not interfere with the viability of keratinocytes, which are highly affected by current antineoplastic therapies^53^. 

Crotoxin provokes possible damage to the cellular DNA of PANC-1 cells, associated with pancreatic tumors, by upregulating protein expression^53^. DNA damage has also been observed in glioma cell lines, leading to an increase in the percentage of cells undergoing apoptosis. Some *in vitro* studies have also reported a higher percentage of apoptosis among SK-MES-1 cells, a lung cancer cell line, in addition to damage such as nuclear condensation and fragmentation^50^ ^,^ ^57^.

When associated with tyrosine kinase inhibitors, crotoxin potentiates the antitumor effect of the drug against lung tumor cell lines^50^ ^,^ ^53^ ^,^ ^57^. In a dose-dependent manner, the toxin prevents DNA synthesis and interrupts the cell cycle in the S phase, suppressing the proliferation of SK-MES-1 cells both *in vitro* and *in vivo* ^52^ ^,^ ^57^. One of the mechanisms identified was the increased expression and cleavage of caspase-3, which is responsible for inducing cell apoptosis^50^ ^,^ ^57^. Another mechanism observed was the induction of cytochrome C release, which increased the occurrence of cellular autophagy, a mechanism also observed in MCF-7 breast cancer lines^47^ ^,^ ^49^ ^,^ ^53^.

Crotoxin also induces the release of LXA4, pro-inflammatory eicosanoid lipoxin, and its analogs through the induction of its synthesis in macrophages^36^ ^,^ ^44^ ^,^ ^46^ ^,^ ^48^ ^,^ ^51^. *In vivo* studies of Walker 256 carcinoma cells concluded that this mechanism is responsible for the antineoplastic action of crotoxin on the lineage, and the concentration of lipoxin was 74% higher in the plasma of animals treated with crotoxin than in those treated with saline solution^51^. Lipoxins have been shown to be antineoplastic owing to their ability to inhibit tumor growth by inhibiting endothelial cell proliferation and reducing the production of angiogenic growth factors^46^ ^,^ ^51^.

Macrophages cultured *in vitro* in the presence of crotoxin secreted 47% less angiogenesis mediators than macrophages from a control group^46^, confirming the role of the toxin in reducing tumor blood vessel neoformation.

The efficacy of crotoxin in dose-dependent inhibition of human esophageal carcinoma tumor growth (Eca-109 cells) was demonstrated *in vivo* ^55^ ^,^ ^57^. The toxin causes cellular damage to the lineage, such as formation of pyknotic cell nuclei, cell lysis, and DNA damage^55^. Exposure of tumor cells to crotoxin also resulted in an increase in the number of stagnant cells in the G1 phase of cell division^53^ ^,^ ^55^ ^,^ ^57^. Increased expression of caspase 3, p17, and p15 proteins and reduced production of Bcl-25 protein can be envolved^55^. 

*In vivo* studies on the HL-60 leukemia cell line showed lower tumor growth inhibitory activity, suggesting that it acts preferentially on solid tumors^21^ ^,^ ^47^ ^,^ ^48^ ^,^ ^50^. The treatment of patients with solid tumors refractory to conventional antineoplastic therapies with the administration of different doses of crotoxin has demonstrated efficacy in reducing different types of carcinomas^21^. Mechanisms of mitochondrial collapse, cytochrome C release, and caspase 3 activation induced cell death in the human leukemia-associated K562 cell line, with the induction of apoptosis and autophagy observed^50^ ^,^ ^57^. 

Crotoxin has been shown to be more cytotoxic than standard chemotherapeutic agents for the treatment of glioma, pancreatic cancer, esophageal cancer, and cervical cancer. Therefore, novel antineoplastic therapies are of great interest, particularly against leukemia, lung cancer, colon cancer, renal cancer, ovarian cancer, esophageal carcinoma, breast carcinoma, melanoma, and brain tumors, whose proliferation is already known to be preventable by the toxin^19^ ^,^ ^53^ ^,^ ^57^. New drugs derived from the toxin, such as VRCTC-310 and CB24, have already been studied in murine and human cell lines^16^ ^,^ ^17^ ^,^ ^21^ ^,^ ^41^ ^,^ ^48^. 

## PHOSPHOLIPASES A_2_

PLA_2_ are type 1 and type 2 enzymes associated with the induction of inflammatory processes, lipid membrane metabolism, and release of substances such as prostaglandins, prostacyclins, thromboxanes, and leukotrienes^16^ ^,^ ^21^ ^,^ ^58^ ^-^ ^60^. 

These enzymes represent the largest family of proteins contained in the venom^23^ ^,^ ^58^, accounting for up to 80% of total proteins^24^. PLA_2_ induces processes such as edema, blockage of neuromuscular junctions, platelet aggregation, and muscle necrosis^21^ ^,^ ^59^. It has a substantial pharmacological interest owing to a wide range of biological actions^60^. Some enzymes have anticoagulant activity through mechanisms of hydrolysis of procoagulant phospholipids, antagonistic effects with coagulant proteins, and interaction with factor X^25^. Cotrim et al. (2011) suggested that PLA_2_ activity is attributable to its actions at different pharmacological sites, which are responsible for platelet aggregation, myotoxicity, and antibacterial activity, as well as anti-inflammatory and neurotoxic effects^58^. 

PLA_2_ has shown anticancer properties by acting on epithelial growth factor receptors (EGFR), reducing the production of tumor necrosis factor, and inhibiting neoplastic growth in lung carcinoma, human breast carcinoma, and leukemia.

The *Cdt* crotoxin and *Naja naja atra* cardiotoxin association has been conducted to develop “VRCTC-310-Onco,” which aims to interfere with the signaling of EGRFs, reduce the production of tumor necrosis factor, and exert cytotoxic action on tumor cells^16^ ^,^ ^48^. The development of EGFR receptor inhibitor drugs represents a new type of therapy against epithelial neoplasms^61^ ^,^ ^62^ given that the receptors act in the signaling responsible for the formation of epithelial cell tumors^61^. 

Reduction in tumor necrosis factor production is also an important mechanism of anticancer action, since the presence of necrosis stimulates tumor phosphorescence mediators, favoring angiogenesis and tumor metastasis.

## GYROXIN

Gyroxin, a member of the serinoprotease family, is a neurotoxic enzyme with coagulant action^4^ ^,^ ^6^ ^,^ ^25^ ^,^ ^45^ ^,^ ^63^, including thrombin-like action^4^ ^,^ ^37^ ^,^ ^45^ ^,^ ^63^ ^,^ ^64^, and represents the second most commonly found family of venoms^37^. Montoni et al. (2020) demonstrated that the toxin also has the ability to cross the blood-brain barrier^35^.

*In vitro* studies have revealed that the enzyme generates clotting in human plasma samples with citrate, with the speed of clot formation being directly proportional to the amount of gyroxin^25^, causing the breakdown of fibrinogen into fibrinopeptide A^25^. Gyroxin is the enzyme responsible for the coagulant activity of Cdt venom as it rapidly consumes circulating fibrinogen, making the blood incoagulable.

Brazilian researchers have used this activity to develop a biopolymer (Heterologous Fibrin Sealant, HFS), which consists of a fibrinogen-rich cryoprecipitate extracted from buffalo blood and a thrombin-like enzyme (gyroxin) purified from *Crotalus durissus terrificus* snake venom^27^ ^,^ ^63^ ^-^ ^65^. They successfully evaluated the safety and immunogenicity of HFS for the first time, estimated the optimum dose, and assessed its preliminary efficacy in the treatment of chronic venous ulcers (CVU) in a phase 2 clinical trial^27^ ^,^ ^63^ ^-^ ^65^.

As gyroxin can cross the blood-brain barrier, it can be an important tool for studies of tumors of the brain and central nervous system.

## CONVULXIN

Convulxin is a high-molecular-mass glycoprotein of the C-type lectin family, which has potent platelet activating and aggregating action^4^ ^,^ ^6^ ^,^ ^66^ ^,^ ^67^, with high affinity for platelets^66^. However, its effect on human peripheral blood mononuclear cells (PBMCs) and the immune system remains unclear.

The mechanism of action of convulxin involves the activation of phospholipase C and its rapid phosphorylation, which is similar to the mechanism induced by collagens in mediating platelet aggregation^66^.

In *in vitro* studies utilizing citrated human plasma samples, the protein generated clot formation without interfering with factors of the coagulation cascade^25^. 

## CROTAMINE

Crotamine is a non-enzymatic polypeptide with myotoxic and neurotoxic actions, responsible for causing cell death in skeletal muscles due to alterations in their sodium channels^4^ ^,^ ^7^ ^,^ ^10^ ^,^ ^18^ ^,^ ^28^ ^,^ ^37^ ^,^ ^68^ ^-^ ^71^. 

A great curiosity is that this myotoxin is not present in all individuals of the species, being thus classified as crotamine-positive or crotamine-negative venom-producing animals^7^ ^,^ ^18^ ^,^ ^23^ ^,^ ^28^ ^,^ ^33^ ^,^ ^34^ ^,^ ^45^ ^,^ ^71^. In crotamine-positive venom-producing animals, the toxin comprises approximately 10%-15% of the venom^31^ ^,^ ^33^ ^,^ ^71^ ^,^ ^72^.

This toxin induces skeletal muscle contraction through its action on sodium channels, interfering with ion permeability in the sarcolemma and reducing the resting potential of the membranes^18^ ^,^ ^28^ ^,^ ^69^. The changes in permeability cause a greater influx of sodium and calcium ions, which are responsible for depolarization, muscle contraction, vacuolization of sarcoplasmic reticulum, rupture of actin and myosin muscle filaments, and muscle necrosis^18^ ^,^ ^28^ ^,^ ^69^ ^,^ ^71^. 

Crotamine displays analgesic, antibacterial, antifungal, antiparasitic, and antitumor actions^4^ ^,^ ^7^ ^,^ ^18^ ^,^ ^26^ ^,^ ^28^ ^,^ ^71^ ^,^ ^73^ ^-^ ^76^. It can be classified as a cell-penetrating peptide, a protein transduction domain, a Trojan peptide, or a membrane translocation sequence^18^ ^,^ ^26^ ^,^ ^28^ ^,^ ^68^ ^,^ ^72^. 

Translocation across cell membranes occurs by binding between crotamine and cell surface heparan sulfate proteoglycans, endocytosis, and accumulation of the toxin in intracellular vesicles^18^ ^,^ ^28^ ^,^ ^69^ ^-^ ^72^ ^,^ ^75^ ^,^ ^76^. To reach the cytoplasm, crotamine induces changes in the permeability of vesicles, causing it to be released and dispersed in the cell^18^ ^,^ ^69^ ^,^ ^72^ ^,^ ^76^. In the cytoplasm, it can bind to centrosomes in the G1 phase of cell proliferation and enables the diagnosis of cell division phases by functioning as a molecular marker^18^ ^,^ ^68^ ^-^ ^71^. 

The antitumor and antimicrobial properties of crotamine are due to its ability to bind to surfaces and acidic cellular compartments such as lysosomes^28^ ^,^ ^74^ ^-^ ^76^. In tumor cells, the prevalence of negatively charged surface molecules, such as phospholipids and mucins, allows preferential binding with the toxin compared to that in healthy cells with electrically neutral surfaces^70^. 

To develop new drugs, synthetic analogs of crotamine were produced, composed of peptides with smaller chains, and were used to study their functions in comparison to natural crotamine, revealing the possibility of producing crotamine derivatives with important antimicrobial and antineoplastic functions^18^ ^,^ ^74^. 

Crotamine possesses preferential selectivity for proliferating cells and for certain phases of the cell cycle^18^ ^,^ ^69^ ^,^ ^71^ ^,^ ^72^ ^,^ ^74^ ^-^ ^77^. Both *in vitro* and *in vivo* studies have demonstrated specific and aggressive cytotoxicity against different tumor types^69^.

The role of crotamine against murine melanoma cells, human melanoma cells, and primary human pancreatic adenocarcinoma cells has been extensively studied^26^ ^,^ ^68^ ^-^ ^71^. Although it is cytotoxic to normal cells when administered at high doses, it is non-toxic at low doses^18^. 

Crotamine administered via the intraperitoneal route at a concentration of one microgram per animal per day for 21 days demonstrated efficacy in reducing tumors in rats with subcutaneous melanoma^68^ ^-^ ^72^. 

Crotamine’s action mechanisms to induce cell apoptosis include the activation of caspases, the reduction of mitochondrial membrane potential, and consequent alteration of organelle membrane permeability, inducing the release of intracellular calcium ions and the influx of extracellular calcium^28^ ^,^ ^68^ ^,^ ^70^ ^,^ ^71^ ^,^ ^73^ ^,^ ^76^. The activation of caspases is one of the mechanisms responsible for cell apoptosis signaling^78^. Its activation can occur by alterations in mitochondrial membrane permeability, which generates cytochrome C release that amplifies apoptosis signals^69^ in HL-60 cells from human leukemia and urinary bladder tumors^69^.

Owing to the ability of the toxin to penetrate cells, it is a potential delivery mechanism for other drugs and antitumor agents^18^ ^,^ ^28^ ^,^ ^68^ ^,^ ^69^ ^,^ ^71^ ^-^ ^74^. In addition to representing a possible antineoplastic or adjuvant therapy, crotamine can be used as a diagnostic marker for cancer^70^ ^,^ ^73^ ^,^ ^76^. Crotamine can be used as a diagnostic marker in human epithelial carcinoma (HeLa), human pancreatic adenocarcinoma (BxPC-3), human breast carcinoma (BT-474), and human colorectal carcinoma (Caco2) cells. 

## L-AMINO OXIDASES (LAAOS)

LAAOs are flavoenzymes responsible for catalyzing amino acids, which generate alpha-keto acids, ammonia, and hydrogen peroxide^14^ ^,^ ^16^ ^,^ ^17^ ^,^ ^21^ ^,^ ^32^ ^,^ ^79^ ^,^ ^80^. Members of this enzyme class are highly toxic and have great pharmacological importance^16^ ^,^ ^79^, as they can cause platelet aggregation, hemorrhage, edema, cytotoxicity, and induction of apoptosis^14^ ^,^ ^16^ ^,^ ^17^ ^,^ ^37^ ^,^ ^79^ ^,^ ^80^.

These enzymes induce apoptosis in human leukemia cells. Their toxicity is mainly attributed to the formation of hydrogen peroxide during oxidative reactions, among other mechanisms^16^ ^,^ ^21^ ^,^ ^30^ ^,^ ^79^ ^,^ ^80^. Although cytotoxic to tumor cells, LAAOs do not affect healthy cells^21^ ^,^ ^80^.

The species-specific cytotoxicity of LAAOcdt was evaluated using nine human cancer cell lines, including pancreatic, esophageal, cervical, and glioblastoma tumors^80^. 

Purified LAAOs can act on different targets of cellular mechanisms such as DNA fragmentation, chromatin condensation, and nuclear fragmentation. Another mechanism is the induction of P53 protein expression, which is synthesized from a tumor suppressor gene that is functionally deficient in more than half of the human tumors^78^ ^,^ ^79^ ^,^ ^81^. Moreover, the induction of protein expression would be relevant to stimulate the monitoring of genome integrity, allowing the identification of damage and repair, resulting in the reduced proliferation of cells with genetic mutations.

## LECTINS

Lectins belong to a family of proteins and glycoproteins that generate platelet aggregation^10^ ^,^ ^16^ ^,^ ^20^ ^,^ ^67^. C-type lectins are non-enzymatic calcium-dependent proteins that affect cell adhesion, endocytosis, and neutralization of pathogens^67^. These proteins may also interfere with tumor proliferation, which has been observed using lectins from venom of other species, offering potential for antineoplastic therapy^16^.

## METALLOPROTEASES

Metalloproteases present hemorrhagic action and induce coagulation alterations in the prey^16^ ^,^ ^21^ ^,^ ^82^. These proteins are copious in crotalid venom^82^ but are present in low quantities in Cdt venom, thus conferring low proteolytic and hemorrhagic activity^33^. 

This class of enzymes is composed of endopeptidases that degrade extracellular matrix proteins, blood components, and endothelial cells^21^. In addition, metalloproteases play a fibrinolytic role and act as prothrombin activators, blood coagulation factor X activators, apoptosis inducers, platelet aggregation inhibitors, pro-inflammatory agents, and inactivators of serinoprotease inhibitors^82^. Different groups of metalloproteases found in viperid and crotalid venoms are involved in tumor proliferation and angiogenesis processes^16^. However, specific studies on Cdt venom metalloproteinases have not yet been conducted.

## DISINTEGRINS

Disintegrins are also important for the inhibition of tumor cells, together with metalloproteases, by acting against angiogenesis and metastasis^16^. This group of non-enzymatic proteins of low molecular mass can interact with integrins expressed by different cells^16^ ^,^ ^17^ ^,^ ^20^ ^,^ ^83^, important cell surface receptors that are involved in interactions between cells and between cells and the extracellular matrix^16^ ^,^ ^17^ ^,^ ^20^ ^,^ ^21^ ^,^ ^83^.

Aggrastat® (Tirofiban, Merck & Co.) and Integrilin® (Eptifibatide, Cor Therapeutics, now part of Millennium Pharmaceuticals) were developed based on snake disintegrins such as echistatin from the saw-scaled viper *Echis carinatus* and barbourin from the southeastern pygmy rattlesnake *Sistrusus miliarius barbouiri* ^14^ ^,^ ^20^ ^,^ ^84^.

Integrins, one of the most important targets of antineoplastic action, are cell surface adhesion molecules that function as receptors and transmitters of cellular signals for migration, invasion and cell proliferation^16^ ^,^ ^83^. Inhibition of integrins is important because it affects cell proliferation, angiogenesis, and metastasis and is a widely studied antineoplastic treatment option^16^ ^,^ ^83^.

Disintegrins isolated from Cdt venom inhibit the interaction between tumor cells, impairing their motility, and preventing the invasion of other tissues^21^. One of the mechanisms involved is the deposition of fibrin around the tumor, which limits its growth.

## PHOSPHODIESTERASES

These enzymes are less abundant in the venom, representing only approximately 2% of its total^33^. Despite being present in the venom in low quantities, it is responsible for inducing important clinical signs of intoxication^33^, and its antitumor activity has not yet been evaluated. 

**FIGURE 2:**
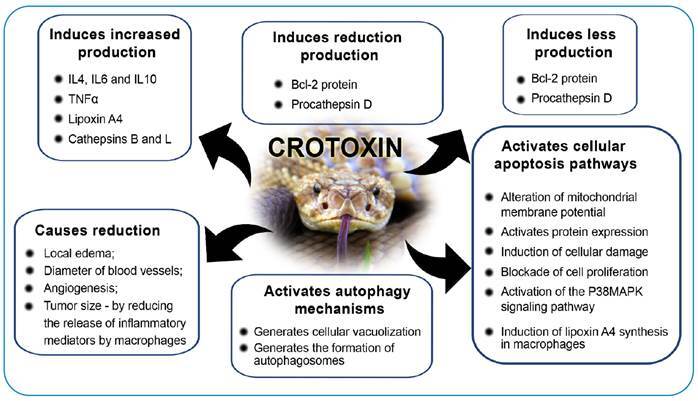
Main antineoplastic actions of *Crotalus durissus terrificus* venom associated with Crotoxin.

**FIGURE 3:**
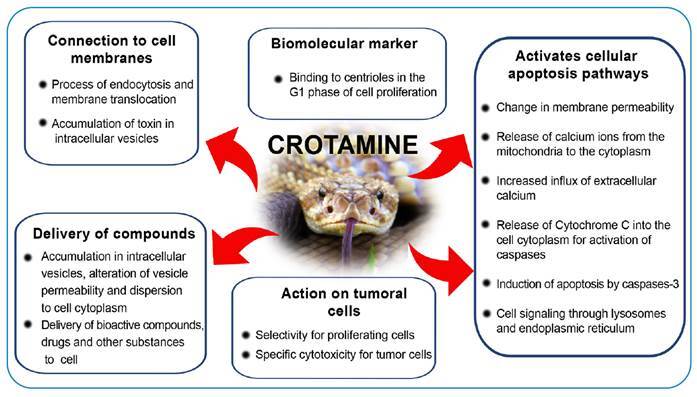
Main antineoplastic actions of *Crotalus durissus terrificus* venom associated with Crotamin.

**FIGURE 4:**
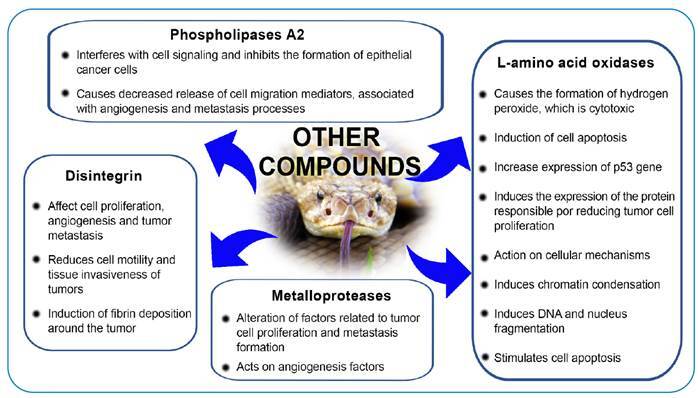
Main antineoplastic actions of *Crotalus durissus terrificus* venom associated with other venom compounds.

Figure 2, Figure 3, and Figure 4 summarize the main mechanisms of antineoplastic action for each component of *Crotalus durissus terrificus* venom*.*

## CLINICAL TRIALS

Crotoxin was administered to patients with solid tumors that were refractory to standard therapy in a phase 1 clinical trial that observed a partial response of more than 50% reduction in tumor mass and a complete response in three of the 23 evaluated patients^21^ ^,^ ^48^ ^,^ ^77^. The authors concluded that crotoxin is a new class of anticancer agents that acts through a novel mechanism of action and thought that neurotoxicity could be the principal toxic effect and appears to be manageable. They recommended 0.18 mg/m^2^ a therapeutic dose for Phase II studies^77^.

The same research team proposed an innovative design for a phase 1 trial with intra-patient dose escalation to study crotoxin^85^. As recorded on the clinical trial platform ClinicalTrial.gov, 18 patients were recruited for this study between 2015 and 2018. The researchers stated that the results would be published shortly^86^.

## CONCLUSIONS

After elucidating the various mechanisms of action of the *C. d. terrificus* venom, it may be stated that this venom is a potential candidate for the development of new antineoplastic therapies that are efficient against different tumor lines and act on different cellular targets.

Considering the selective cytotoxicity of venom components for tumor cells to the detriment of healthy cells, the development of innovative therapies against cancer, based on the bioactive compounds of the rattlesnake, may present greater benefits compared to current therapeutic protocols, such as chemotherapy and radiotherapy, which are known to cause alterations in the normal cells of cancer patients. 

The therapeutic use of compounds from *Crotalus durissus terrificus* snake venom also represents an alternative for the treatment of tumors resistant to drugs currently available on the market.

Therefore, one can conclude that the improvement of studies of the different fractions of ophidian venom is of great pharmacological interest, with potential for immense impact on the future of therapeutic medicine. 
